# Metformin and LW6 impairs pancreatic cancer cells and reduces nuclear localization of YAP1

**DOI:** 10.7150/jca.33029

**Published:** 2020-01-01

**Authors:** Xianbin Zhang, Peng Liu, Yuru Shang, Hagen Kerndl, Simone Kumstel, Peng Gong, Brigitte Vollmar, Dietmar Zechner

**Affiliations:** 1 Institute for Experimental Surgery, Rostock University Medical Center, Schillingallee 69a, 18059, Rostock, Germany;; 2 Shandong Cancer Hospital and Institute, Shandong First Medical University and Shandong Academy of Medical Sciences, Jiyan Road 440, 250117, Jinan, China;; 3 Department of General Surgery, Shenzhen University General Hospital, Xueyuan Road 1098, 518055, Shenzhen, China;; 4 Molecular Oncology and Immunotherapy, Department of General Surgery, Rostock University Medical Center, Schillingallee 69, 18059, Rostock, Germany.

**Keywords:** Metformin, LW6, YAP1

## Abstract

The poor survival rate of pancreatic cancer is still a major challenge for the clinicians and their patients. In this study, we evaluated the efficacy of metformin, an inhibitor of oxidative phosphorylation, in combination with LW6, which impairs malate dehydrogenase 2 activities, in treating pancreatic cancer cells. We observed that this combinational therapy significantly reduced cell proliferation, migration, and significantly induced cell death when compared to cells treated by each monotherapy or Sham. In addition, we found that the combination of metformin and LW6 increased the phosphorylation of yes-associated protein 1 at serine 127 and attenuated the nuclear localization of this transcription factor. This combinatorial treatment also decreased the level of cellular yes-associated protein 1. This suggests that metformin in combination with LW6 impairs pancreatic cancer cells and reduces nuclear localization of yes-associated protein 1.

## Introduction

In 1997, Burris et al. reported that gemcitabine could modestly improve overall survival (5.65 months vs. 4.41 months), when compared to fluorouracil (5-FU), in metastatic pancreatic cancer patients 1. Subsequently, gemcitabine became the first-line chemotherapy for pancreatic cancer 2, 3. Unfortunately, local recurrence and distant metastasis still occur in 41% of those patients, who underwent curative resection and were treated with gemcitabine 4. In addition, the 5-year survival rate of advanced pancreatic cancer patients is just 3% 5. Thus, there is still a need to explore novel treatment strategies for pancreatic cancer patients.

Metformin, an inhibitor of oxidative phosphorylation, is considered as an emerging therapy to impair several cancers 6-8. Unfortunately, two randomized controlled trials proved that metformin failed to improve the anti-tumor efficacy of traditional chemotherapeutics, such as gemcitabine, cisplatin and capecitabine, in pancreatic cancer patients 9, 10. In addition, our previous study proved that metformin could inhibit the gemcitabine-induced apoptosis in pancreatic cancer cells 11. This suggests that researchers should evaluate the anti-tumor efficacy of metformin in combination with novel drugs rather than traditional chemotherapeutics.

Recently, it has been demonstrated that LW6, a novel inhibitor of malate dehydrogenase 2, can impair the mitochondrial tricarboxylic acid cycle 12, 13 and inhibit proliferation of liver cancer cells 14. However, it was still unclear if and how LW6 impairs pancreatic cancer cells.

Yes-associated protein 1 (YAP1) is a master component of the Hippo-YAP1 signaling pathway in mammals 15. Evidence proved when this pathway is switched “OFF”, YAP1 can translocate to nucleus and induce the expression of several genes, such as *cysteine-rich 61 (CYR61)* 16 and *connective tissue growth factor (CTGF)* 17, which induce the migration and proliferation of cancer cells 15, 18, 19. However, when cells are under energy stress, this pathway is switched “ON”, YAP1 is phosphorylated at serine 127 and retained in the cytoplasm 15, 18, 19. Moreover, YAP1 can also be phosphorylated at serine 381 or serine 384, which triggers degradation of this protein 20. In both cases YAP1 cannot move to the nucleus to promote the transcription of its target genes 15, 18, 19. In addition, more and more clinical studies documented that YAP1 is highly expressed in pancreatic tumor and that its expression is associated with poor survival 21, 22. This suggests that impairing nuclear YAP1 accumulation may be a promising therapeutic strategy to treat pancreatic cancer.

Thus, the purpose of this study was to evaluate the anti-cancer efficacy of metformin plus LW6 in pancreatic cancer cells. In addition, the present study also aimed to assess if YAP1 is involved in the anti-cancer effect of metformin plus LW6.

## Materials and Methods

### Cell culture and reagents

The murine pancreatic adenocarcinoma cell line, 6606PDA (a gift from Prof. Tuveson at the University of Cambridge, UK), and the human pancreatic cancer cell line, PANC-1 (purchased from ATCC, Manassas, USA), were cultured in Dulbecco's Modified Eagle's Medium (DMEM) (Biochrom, GmbH, Berlin, Germany, code FG 0435) supplemented with 10% fetal calf plasma (FCS), 100 units/ml penicillin and 100 μg/ml streptomycin. Metformin (code D150959) and mitomycin C (MCC, code M7949) were purchased from Sigma-Aldrich (St. Louis, USA) and dissolved in phosphate buffered saline (PBS). LW6 was purchased from Merck Millipore (Eschborn, Germany, code 400083) and dissolved in dimethyl sulfoxide (DMSO). Lysophosphatidic acid (LPA), which induces cell migration via increasing the accumulation and nuclear localization of YAP1 23, was purchased from Enzo Biochem (Farmingdale, New York, USA, code BML-LP100-0005) and dissolved in PBS. All these solutions were stored at -20°C. In addition, cyano-4-hydroxycinnamat (CHC) was obtained from Tocris Bioscience (Bristol, UK, code 5029), and CPI-613 (CPI) was purchased from Hölzel Diagnostika (Cologne, Germany, code M2297).

### Evaluation of proliferation, cell death and migration

To evaluate proliferation, 2×10^3^ 6606PDA cells per well were seeded in a 96 well plate. After 24 hours, these cells were treated with medium containing DMSO (Sham) or therapeutic agents as indicated in each figure for 48 hours. Subsequently, the proliferation of cells was evaluated by quantifying the incorporation of the 5-bromo-2'-deoxyuridine (BrdU) with the colorimetric Cell Proliferation ELISA kit (Roche Diagnostics, Mannheim, Germany, code 11647229001) and Perkin Elmer Victor X3 model 2030 Multilabel Plate Reader platform (PerkinElmer, Waltham, USA).

**Figure 1:**
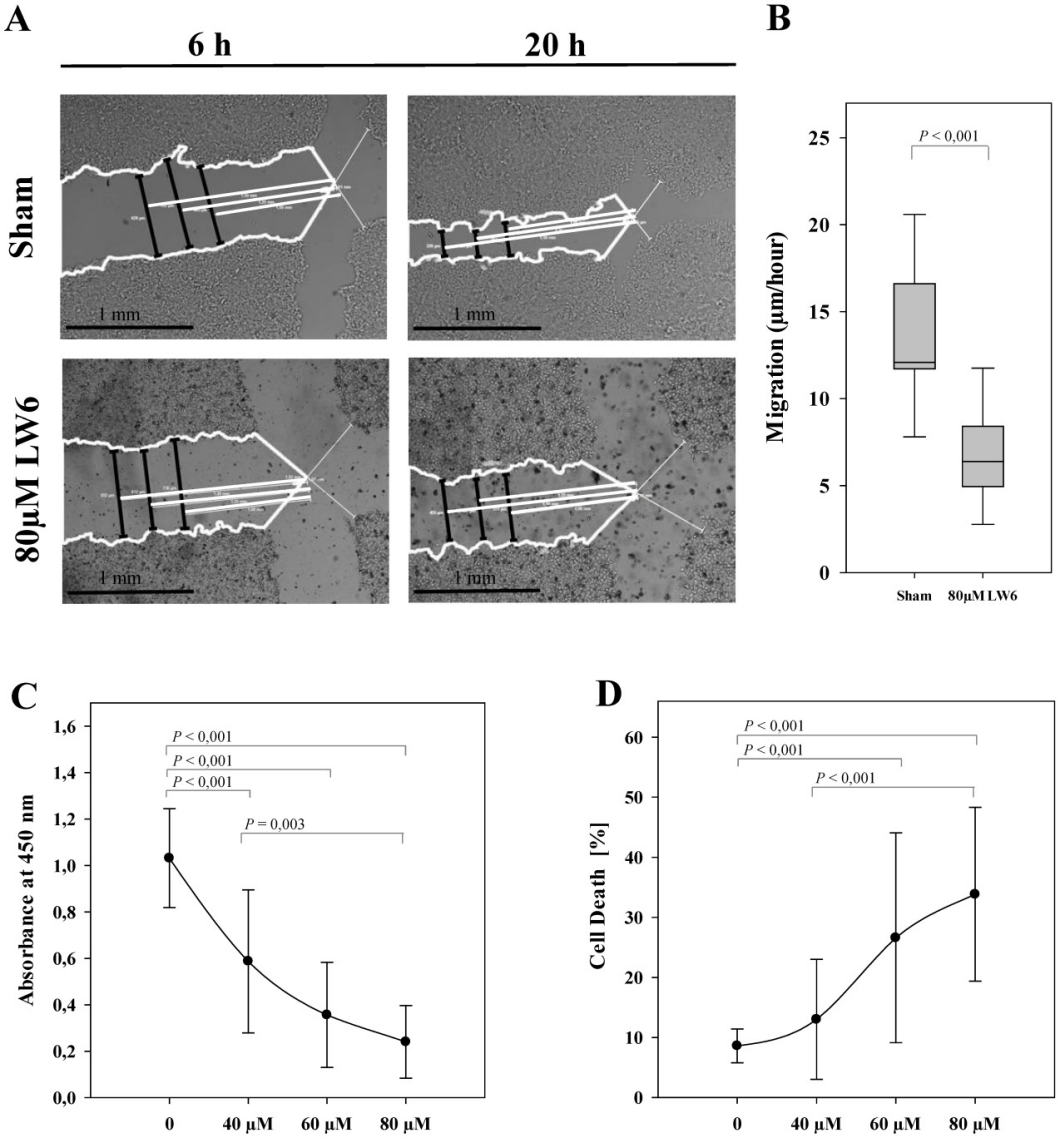
** LW6 inhibits migration, proliferation and viability of pancreatic cancer cells.** After scratching the cell monolayer, vehicle solution (Sham) or 80 µM LW6 was added and the distance of the gap (black lines) was measured at three distinct locations with the help of three white reference lines (at 1.00 mm, 1.25 mm and 1.50 mm distance from the middle of the scratch) at 6 hours and 20 hours (A). LW6 significantly inhibited migration of 6606PDA cells when compared to cells treated with vehicle solution (B). In addition, LW6 inhibited cell proliferation (C) and induced cell death (D) in a dose dependent manner. n = 18 per group for B, n =14 per group for C, and n = 12 per group for D. Bar = 1 mm.

**Figure 2:**
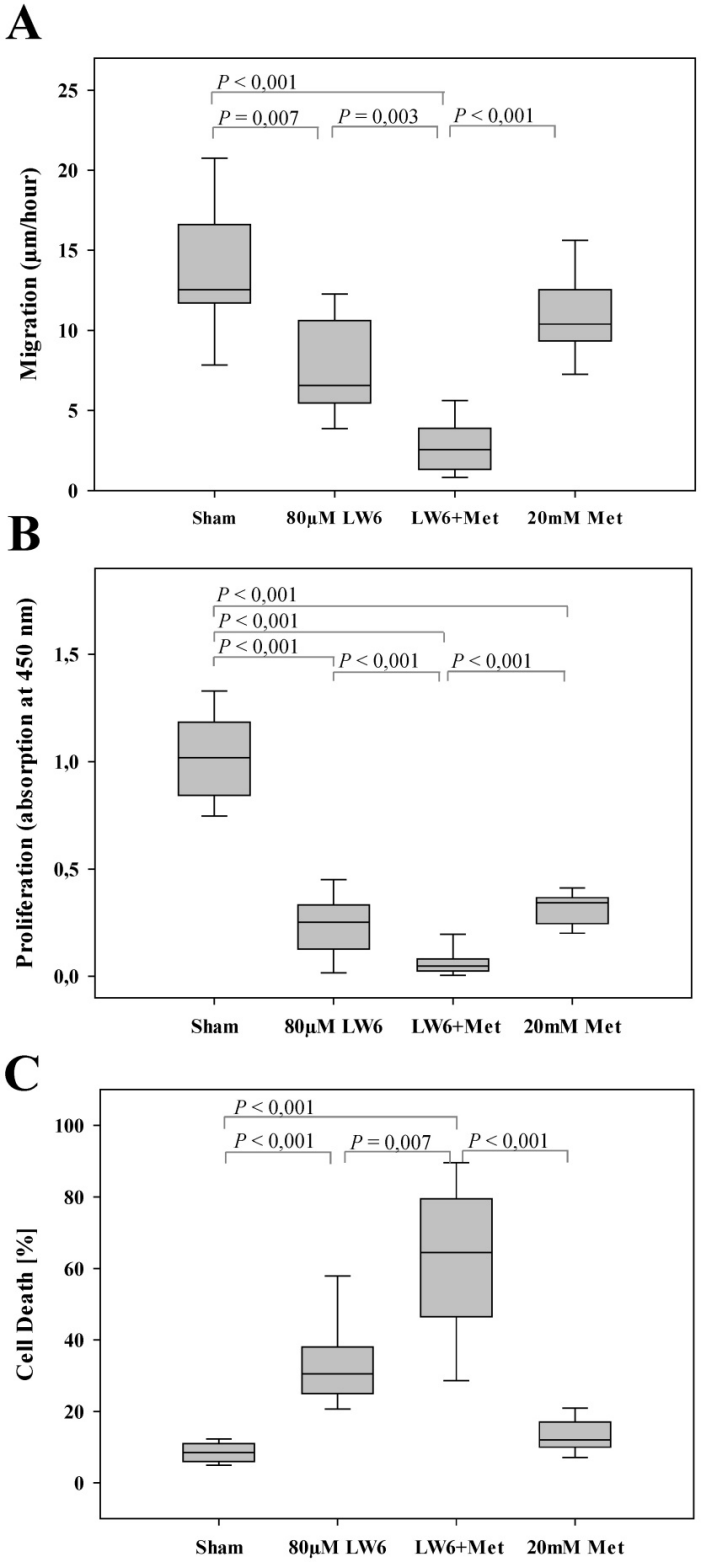
** LW6 enhances the anti-cancerous efficacy of metformin.** Metformin modestly inhibited migration of 6606PDA cells, whereas LW6 in combination with metformin (Met) significantly reduced cell migration when compared to each monotherapy or Sham treatment (A). In addition, LW6 enhanced the anti-proliferation efficacy of metformin (B) and metformin-induced cell death (C). n = 10 per group for A, n =14 per group for B, and n = 12 per group for C.

In order to analyze cell death, 3×10^4^ 6606PDA per well were plated in a 24 well plate. On the following day, these cells were treated with the appropriate vehicle (Sham), LW6, metformin or both drugs. The used concentration of each drug for each experiment is indicated in Figure 1 and Figure 2. After treating for 54 hours, trypan blue assays were performed. The percentage of dead cells was determined with the help of a Neubauer chamber in a blinded fashion.

To evaluate cell migration, 1×10^6^ 6606PDA cells per well were seed in a 6 well plate. On the following day, these cells were allowed to grow until they reached 100% confluency. Subsequently, they were treated with 10μg/ml MMC for 3 hours to stop the proliferation. After washing cells with PBS carefully, the cell monolayer was scratched and these cells were treated with the appropriate vehicle (Sham), 80µM LW6, 20mM metformin, 2.5µM LPA or combinations of drugs as indicated in each figure. The distance of the gap was measured at three distinct locations as indicated in Figure 1A, with a Leica microscope, DMI 4000B, and a suite software (Leica Mikrosysteme Vertrieb GmbH, Wetzlar, Germany), after treating cells for 6 hours and 20 hours. Subsequently, the mean speed of migration was determined as following: speed = [(mean distance of three locations at 6 hours - mean distance of three locations at 20 hours) / (20 hours - 6 hours)] / 2 (to adjust for migration from both sides) 24. In order to investigate, if overexpression of phosphorylation deficient YAP1 impairs the anti-migration effect of LW6 and metformin, 2×10^5^ 6606PDA cells were seeded in a ThinCert TM cell culture insert (Greiner Bio-One, Leipzig, Germany, code 662638) and cultured in a 24 well plate in the absence of FCS for 24 hours. Subsequently, these cells were transfected with the p2xFLAGhYAP1-S127A plasmid (Addgene, Cambridge, UK, code 17790), a kind gift from Marius Sudol 25, with the help of Lipofectamine 3000 (Thermo Fisher Scientific, Waltham, USA, code L3000001), and treated by 80µM LW6 in combination with 20mM metformin in the presence of FCS for 40 hours. The migratory cells were determined by 1% crystal violet staining solution (Sigma-Aldrich, St. Louis, USA, code V5265) and the absorption was measured at 750nm by a Tecan Infinite 200 Microplate Reader (Tecan, Männedorf, Switzerland).

### Evaluation of YAP1

To determine the level and localization of YAP1 in cells, 2.4×10^5^ 6606PDA cells were seeded in a glass bottom dish (NEST, Wuxi, China, code 801001). After 24 hours, these cells were treated with DMSO (Sham), LW6, and LW6 plus metformin for 6 hours. Subsequently, they were fixed with 4% formalin, permeabilized with 0.1% Triton X-100, and treated with 2.5% Bovine Serum Albumin (BSA). Then these cells were incubated with YAP1-antibody (Novus Biologicals, Littleton, Colorado, USA, code NB110-58358) for 1.5 hours at room temperature, followed by the second antibody, Alexa Fluor 488 (Sigma-Aldrich, St. Louis, USA, code A-11070) for 1 hour. The nuclei were stained with 4'6-diamidino-2-phenylindole (DAPI). Images were acquired by a confocal microscope, Zeiss LSM 780 (Zeiss, Oberkochen, Germany), using the 60× oil objective. The average intensity of nuclear YAP1 was determined with the help of Adobe Photoshop CS5 software (Adobe Systems Inc, San Jose, California).

In order to quantify the cellular concentration of YAP1 and the phosphorylation on serine 127 of YAP1, 2.4×10^5^ 6606PDA or PANC-1 cells per well were plated in a 6 well plate. Western blot was performed as previously described 24 with the following antibodies: rabbit anti-YAP1 (Novus Biologicals, Littleton, Colorado, USA, code NB110-58358, dilution: 1000×), rabbit anti-serine 127 YAP1 (Cell Signaling, Danvers, USA, code 4911, dilution: 1000×), mouse anti-β-actin (Sigma-Aldrich, St. Louis, USA, code A5441, dilution: 20000×), rabbit anti-Lamin A+C (Abcam, Cambridge, United Kingdom, code ab133256, dilution: 10000×), rabbit anti-GAPDH (Proteintech, Chicago , USA, code 10494-1-AP, dilution: 80000×), peroxidase linked anti-rabbit (Cell Signaling, Danvers, USA, code 7074, dilution: 10000×) or peroxidase-linked anti-mouse (Sigma-Aldrich, St. Louis, USA, code A9044, dilution: 60000×). The ratios of total YAP1 / β-actin and phosphorylated YAP1 / total YAP1 were determined using a Chemi-Doc XRS System (Bio-Rad Laboratories, Munich, Germany). To evaluate the level of nuclear YAP1, the cell fractionation assay was performed using NE-PER Kit (Thermo Fisher Scientific, Waltham, USA, code: 78833). The level of YAP1, Lamin A+C, and GAPDH were measured on separate gels using the identical extracts.

### Statistical analysis

All statistics were performed by Sigmaplot 12.0 (Systat Software, San Jose, CA, USA). The significance of differences was determined by Mann-Whitney rank-sum test with Bonferroni correction: For multiple testing, the level of statistical significance α = 0.05 was adjusted by the number of comparisons.

## Results

### LW6 inhibits migration, proliferation and viability of pancreatic cancer cells

To evaluate the anti-cancer efficacy of LW6, migration, proliferation, and cell death were analyzed in 6606PDA cells. We observed that 80 µM LW6 significantly inhibited migration (Figure 1A and B). In addition, LW6 reduced cell proliferation (Figure 1C) and induced cell death (Figure 1D) in a dose dependent manner.

### LW6 plus metformin impairs pancreatic cancer cells

In order to evaluate the efficacy of the combinational therapy, metformin plus LW6, we treated pancreatic cancer cells with the appropriate vehicle (Sham), 80 µM LW6, 20 mM metformin, or 80 µM LW6 plus 20 mM metformin. We observed that metformin only moderately reduced cell migration, whereas LW6 significantly inhibited the migration of cells (Figure 2A). Interestingly, the combinatorial treatment, LW6 plus metformin, significantly reduced cell migration when compared to Sham treated cells or when compared to the treatment with single drugs (Figure 2A). In addition, we observed that metformin or LW6 treatment significantly inhibited the proliferation of cells (Figure 2B). Moreover, the combinatorial treatment, LW6 plus metformin, significantly reduced cell proliferation when compared to Sham treated cells or when compared to the treatment with single drugs (Figure 2B). When analyzing the death of cells, we observed that metformin slightly induced cell death, whereas treatment with LW6 lead to a strong and significant increase in the percentage of dead cells (Figure 2C). Interestingly, we observed that LW6 plus metformin significantly induced cell death, when compared to Sham treated cells or when compared to the treatment with each single drugs (Figure 2C). Thus, metformin mainly inhibits proliferation, whereas LW6 significantly inhibits migration as well as proliferation and induces cell death. The combination of LW6 plus metformin impairs the pancreatic cancer cells more efficiently than treatment with a single drug.

### Synergistic effect of LW6 and metformin on YAP1

**Figure 3:**
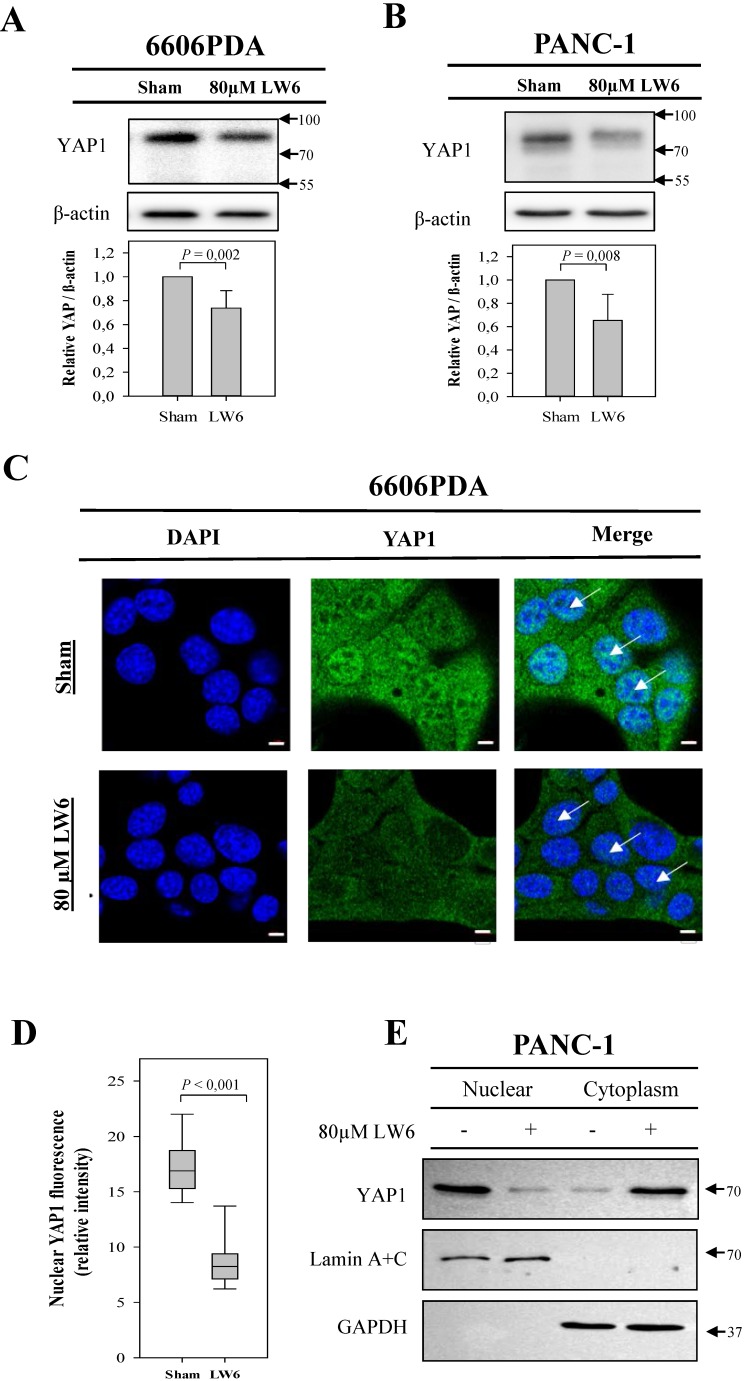
** LW6 attenuates the accumulation of cellular YAP1 and its nuclear location.** After treating 6606PDA cells (A) or PANC-1 cells (B) with LW6 for 12 hours, LW6 decreased the accumulation of YAP1 when compared to Sham treated cells. In addition, after treating 6606 PDA with LW6 cells for 6 hours, the accumulation of YAP1 in the nucleus was attenuated when compared to Sham-treated cells (C and D). Moreover, after incubation of PANC-1 cells with 80 µM LW6 for 12 hours, LW6 decreased the accumulation of nuclear YAP1 (E). n = 6 per group for A, n =5 per group for B, n = 10 per group for D, n = 2 per group for E. Bar = 5µm. Arrows point at nuclei.

**Figure 4:**
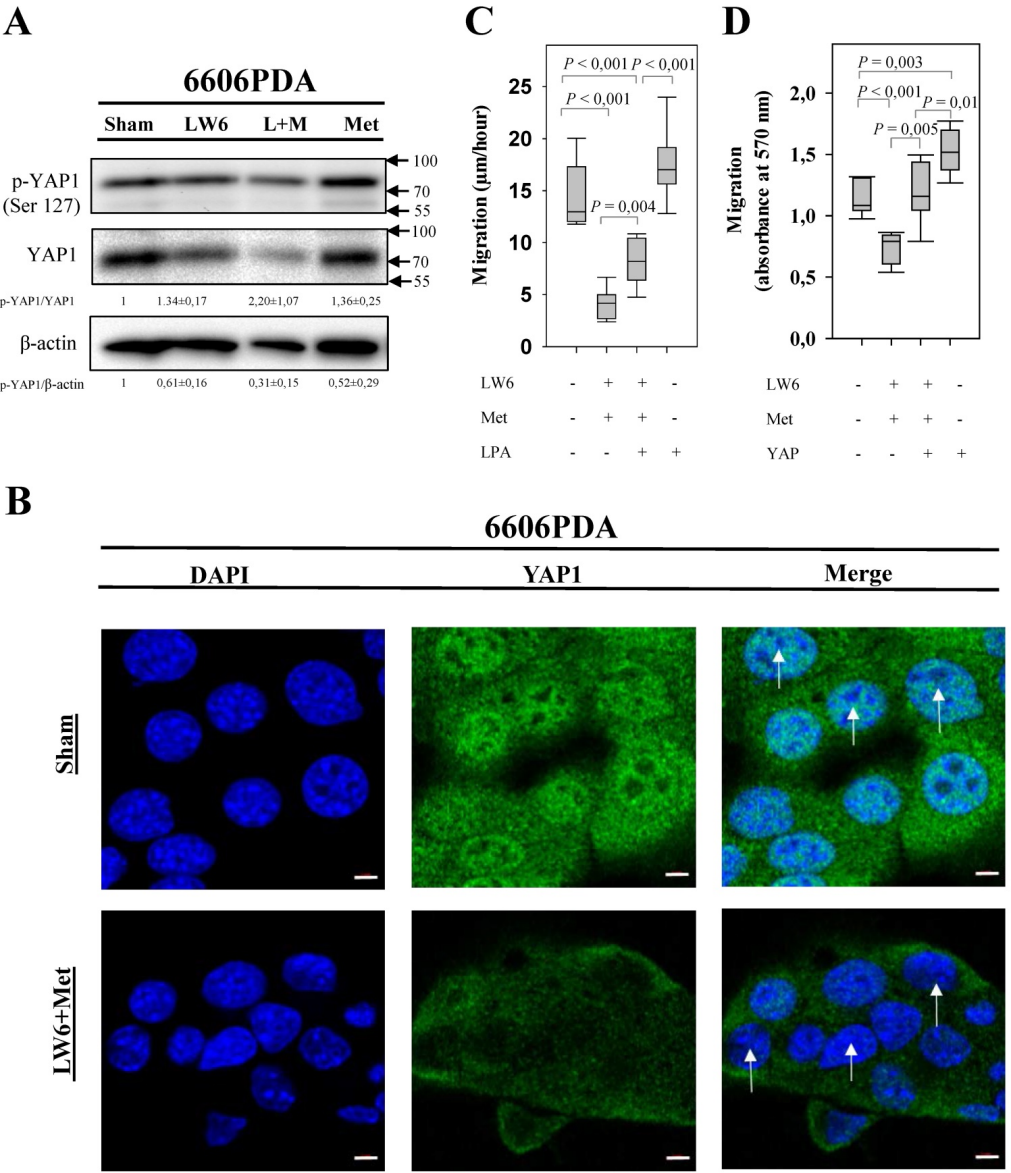
** Synergistic effect of LW6 and metformin on YAP1.** 80 µM LW6, 20 mM metformin (Met) and the combinational treatment metformin plus LW6 increased phosphorylation of YPA1 at serine 127 and decreased cellular YAP1 concentration after treating cells for 24 hours (A). Moreover, this combinational therapy attenuated the nuclear localization of YAP1 compared to Sham treated cells (B). In addition, lysophosphatidic acid (LPA) and the phosphorylation deficient mutant YAP1-S127A stimulate cell migration of 6606PDA cells (C and D). n = 2 per group for A, n = 3 per group for B, n = 7 per group for C, n = 9 per group for D. Bar = 5µm. Arrows point to nuclei.

**Figure 5:**
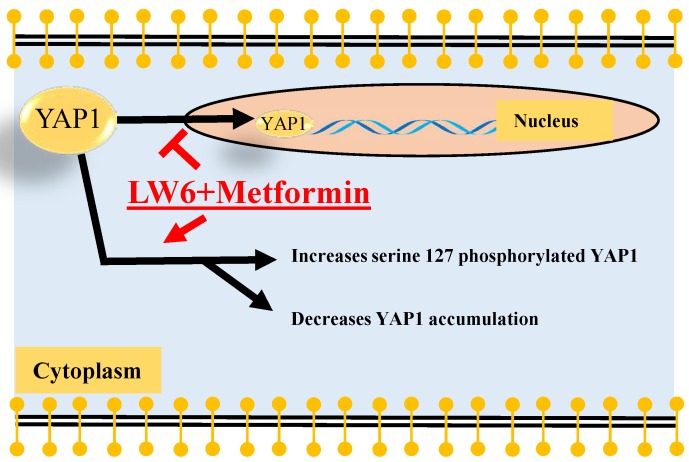
** Summary.** The present study demonstrated that LW6 in combination with metformin enhanced phosphorylation of YAP1 at serine 127, decreased the accumulation of cellular YAP1. Possibly, both processes, cytoplasmic retention and protein degradation might attenuate the nuclear localization of YAP1.

In order to assess if LW6 has an effect on YAP1, we treated pancreatic cancer cell lines with 80 μM LW6 and assessed the protein concentration and nuclear localization of YAP1. We observed that LW6 significantly decreased YAP1 concentration in the cell extract of 6606PDA and PANC-1 cells (Figure 3A and B). Moreover, LW6 treatment prevented nuclear localization of this protein, when compared to Sham treated cells in 6606PDA cells (Figure 3C and D) and PANC-1 cells (Figure 3E). In addition, we observed that LW6 phosphorylated YAP1 at serine 127 and decreased YAP1 concentration (Figure 4A). Treatment with 20 mM metformin also caused phosphorylation of YAP1 and decreased the concentration of this protein in the cell extract (Figure 4A). Interestingly, the combinational treatment had a stronger effect on the phosphorylation and concentration of YAP1 than each of the single drugs. In addition, this combinational therapy attenuated the nuclear localization of YAP1 compared to Sham treated cells (Figure 4B). To evaluate, if YAP1 regulates migration, we applied LPA, which increases nuclear YAP1 23, or transfected the 6606PDA cells with the p2xFLAGhYAP1-S127A plasmid, which expressed a phosphorylation deficient YAP1 protein. We observed that LPA or overexpression of YAP1-S127A stimulates migration, and that LW6 plus metformin inhibits migration even when YAP signaling is activated by LPA or YAP1-S127A overexpression (Figure 4 C and D). Thus, these data demonstrate that YAP1 induces cell migration. In addition, metformin in combination with LW6 increases phosphorylation of YAP1 at serine 127 and impairs the nuclear localization of YAP1 (Figure 5).

## Discussion

The present studies proved that metformin significantly inhibited cell proliferation (Figure 2B) while it only moderately inhibited migration (Figure 2A) and moderately induced cell death (Figure 2C). Significant inhibition of cell proliferation by metformin is observed in many other cell lines 26-28. Moreover, moderate 29 as well as significant 30 inhibition of migration has also been observed in other studies. The regulation of cell death by metformin is more controversial. Some studies demonstrate that metformin moderately induces cell death 27, 31. However, other studies demonstrate that metformin can also significantly inhibit cell death, which is induced by distinct chemotherapeutical agents or oxidative stress 11, 32, 33. This suggests that metformin should only be combined with well selected drugs. LW6 seems to be an ideal combination for metformin, because it inhibits proliferation as well as migration and increases cell death in pancreatic cancer cells (Figure 1) as well as other cells 14, 34-37. Interestingly, we noticed that LW6 significantly improves cell sensitivity to metformin (Figure 2). These data suggest that the combinatorial therapy, LW6 plus metformin, might be a promising strategy to treat pancreatic cancer. This is in contrast to other options, such as gemcitabine in combination with metformin 11, 38. We also evaluated the efficacy of LW6 in combination with other metabolism inhibitors, such as CHC 39 and CPI 40. Unfortunately, we observed that both CHC and CPI significantly impair the anti-proliferative effect of LW6 (Figure S1A and B). This suggests that not in all cases the combination of metabolic inhibitors leads to increased inhibition of proliferation. This supports the old-fashioned approach, to evaluate combinatorial therapies *in vitro* to screen for the best option first, before one starts to do *in vivo* experiments.

When evaluating the feasibility of testing this drug combination on animals or humans the dose and potential toxic side effects have to be considered. Our study demonstrates a partial inhibition of proliferation and moderate induction of cell death at 20 mM metformin (3312 mg/L). Moreover, several pre-clinical studies demonstrated that treating mice with a high dose of metformin, such as 125 mg/kg 25, 41 and 250 mg/kg 41, can successfully decrease pancreatic tumor weight. Considering that the blood volume of mice in milliliter is approximately 8% of their body weight in grams, these mice would have a hypothetical concentration of metformin in the blood of approximately 1562 to 3125 mg/L. This is a dosage similar to the dosage used in our study. However, clinical trials have been conducted using a much lower dosage. For example, Kordes et al. performed a randomized controlled trial to evaluate the benefit of metformin plus standard systemic therapy 9 in advanced pancreatic cancer patients. In their study, metformin was administered 500 mg to 1000 mg twice a day. We speculate that the mean body weight of advanced pancreatic cancer patients is 60 kg 42. Thus, in Korves's study, these patients were treated with 16.7 to 33.4 mg/kg/day metformin, a dosage that is approximately 7.5 fold lower than in most animal experiments. Indeed, metformin failed to improve the survival time of pancreatic cancer patients in this clinical study 9. Notably, the U.S. FDA approved safe dosage of metformin is 2550 mg (approximately 42.5 mg/kg body weight) daily 9, 43. Possibly a higher dose of metformin might be necessary for treating cancer in animal experiments as well as in patients. Since a higher dose of metformin can cause several adverse effects, such as diarrhea, nausea, and fatal hypoglycemia 43, it has to be carefully evaluated, if possible beneficial effects for cancer patients, justify these adverse effects. Unfortunately, there are only few data, which help to judge a reasonable dosage for LW6. Lee et al. reported that 20 mg/kg LW6 significantly inhibited tumor growth in mice 44. However, they did not analyze toxicological side effects. Thus, future studies need to determine if 20 mg/kg LW6 and if 125-250 mg/kg metformin in combination with 20 mg/kg LW6 is safe and effective in animals and cancer patients.

Since YAP1 is involved in tumorigenesis and metastasis 45, 46, we evaluated the hypothesis if metformin and LW6 have an effect on YAP1. Consistent with one previous study 47, we observed that metformin promotes phosphorylation of YAP1 at serine 127, which leads to 14-3-3 binding and cytoplasmic retention 48. This effect of metformin can be explained by the well-known fact that metformin can activate 5'AMP-activated protein kinase (AMPK) 49, which enhances phosphorylation of YAP1 at serine 127 47. Moreover, we observed that metformin reduced the accumulation of YAP1. This is also supported by a previous study using primary mouse hepatocytes 47. These data suggest that metformin might cause phosphorylation of YAP1 at other serine residues, such as serine 381, and can therefore enhance YAP1 degradation 20. It is well characterized that processes, cytoplasmic retention as well as protein degradation, can attenuate nuclear localization of YAP1 15. In addition, we observed that LW6, the inhibitor of malate dehydrogenase 2, reduces YAP1 accumulation and nuclear localization (Figure 3). LW6 might affect YAP1 by causing an energy crisis. Consistent with this hypothesis, Lee et al. reported that LW6 could inhibit the mitochondrial tricarboxylic acid cycle and reduce ATP production 50. In addition, DeRan et al. found that energy stress could induce YAP1 cytoplasmic retention and serine 127 phosphorylation 51. This might prevent YAP1 from entering the nucleus and may inhibit the transcription of oncogenic genes, such as *CTGF* and *CYR61* 16, 17.

Our data demonstrate that metformin and LW6 can be combined to efficiently inhibit migration and proliferation and to induce cell death, but that these drugs also have a common target: YAP1. Both drugs increase the phosphorylation of YAP1 at serine 127 and decrease the cellular accumulation of YAP1. Surprisingly, we observed that LW6 plus metformin inhibits migration even when YAP signaling is activated by YAP1-S127A overexpression. Thus, these data suggest that LW6 plus metformin might not only target YAP signaling, but also other signaling pathways that regulate cell migration. In conclusion, these data demonstrate that metformin in combination with LW6 impairs pancreatic cancer cells and inhibits nuclear localization of YAP1. Thus, metformin in combination with LW6 is a promising treatment strategy for pancreatic cancer. However, future studies are needed to evaluate the optimal dose of metformin and LW6 for preclinical and clinical studies.

## Supplementary Material

Supplementary figures.
