# Supplementary material for: Metformin and LW6 impairs pancreatic cancer cells and reduces nuclear localization of YAP1
Source: J Cancer. 2020 Jan 1;11(2):479–87. doi: 10.7150/jca.33029 (PMC6930432; doi:10.7150/jca.33029)

Figure S1. α-cyano-4-hydroxycinnamate (CHC) and CPI-613 (CPI) impairs the anti-proliferation effect of LW6. After treating cells with LW6 plus CHC (A) or LW6 plus CPI (B) for 48 hours, we observed that both CHC and CPI significantly impaired the anti-proliferation effect of LW6. n = 9 per group for A and B.

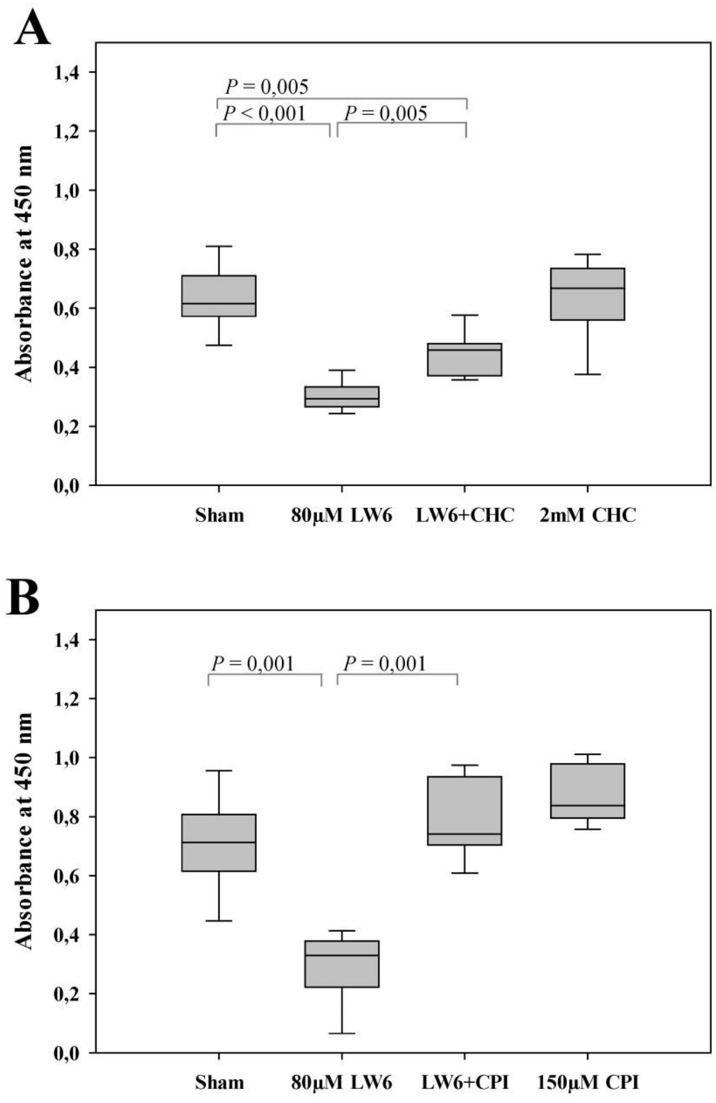

Supplement: Supplementary file 1 — Supplementary figures. [file jcav11p0479s1.pdf]
